# A cross-cultural mixed-methods approach to the conceptualisation of domestic violence in the United Kingdom and Peru

**DOI:** 10.1080/13218719.2024.2441800

**Published:** 2025-02-03

**Authors:** Elsa Rivera, Aleksandra Monteiro

**Affiliations:** Department of College of Health, Psychology, and Social Care, University of Derby, Derby, UK

**Keywords:** cross-culture, domestic violence, intimate partner abuse, Latino studies, mixed-methods

## Abstract

The lack of cross-cultural research on Domestic Violence (DV) for Latinx populations has left a disparity in culturally-informed frameworks and guidelines for best practice. This study proposes that understanding cultural constructs of DV contributes to the formulation of culturally-relevant programmes. Forty-one participants (21 from the UK and 20 from Peru) responded to a mixed-methods designed study that examined the definition of DV and blame attribution, using the Domestic Violence Blame Scale (DVBS). Results revealed the Peruvian public had significantly higher levels of blame attribution in societal, situational and victim blaming than the UK public. However, both the UK and Peru had aligning perpetrator blame attitude scores. This study also found crucial differences when defining DV, its associated characteristic behaviours, and the perception of who can be affected by DV. These findings could be implemented into existing practices to account for the needs of Latinx populations living in the UK.

## Overview of Findings

- The collectivistic and individualistic natures of the Peruvian and British cultures impact the conceptualisation of domestic violence (DV).
- For Peruvians the notion of home and family is central to how and where DV occurs.
- Cultural norms, the acceptance of *machismo,* and *marianismo* as a feminine model seem to affect DV blame attributions.

## Introduction

The global prevalence of intimate partner violence (IPV) varies widely. European countries typically report IPV rates of 10–24%, while South American countries see domestic violence (DV) prevalence ranging from 15% to 40% (SOS Children’s Villages, 2020). In the UK, 2.3 million adults experienced non-sexual domestic abuse in the past year, accounting for 3.4% of the population (Office for National Statistics, ONS, 2020). Around 200,000 DV incidents were reported in 2019, accounting for 5.1% of the Peruvian population (SOS Children’s Villages, 2020). The UK and Peru both face reporting barriers (Cripe et al., 2015; Evans & Feder, 2016), but distinct cultural and legal factors contribute to different IPV prevalence and reporting patterns (Sardinha et al., 2022).

The lack of congruence in legal definitions of IPV across the world is alarming. Cultural ideologies, values and social norms play a critical role in how DV is understood, addressed and experienced. Recognising cultural differences is essential in developing informed interventions where Western frameworks may not be universally applicable across diverse populations. The World Health Organization (WHO; 2024) defines IPV as harm by an intimate partner. UK law defines DV as controlling, threatening or violent behaviour, irrespective of gender or sexuality (UK Government, 2021). The closest legal definition for DV in Peru is the ‘Law of Family Protection’, which includes harm between spouses or co-inhabitants but lacks specificity regarding forms of violence, including psychological abuse (McKenzie, 2023; Women’s Rights Division, 2000).

Cultural and legal definitions collectively contribute to statistical disparities (SOS Children’s Villages, 2020), emphasising the need for nuanced exploration of DV conceptualisations and attitudes in different cultures. One key difference lies in the relationship between the victim and the perpetrator. According to the UK’s legal definition (UK Government, 2021), controlling and coercive behaviour can manifest in various partner relationships, and it offers clear examples of this. In contrast, Peruvian family law centres around marriage and cohabitation (Women’s Rights Division, 2000). Peru has separate laws on sexual harassment to account for sexually-based violence that is not included in its Family Laws (Law no. 27942 cited in McKenzie, 2023; Law no. 30314 cited in McKenzie, 2023). However, this excludes married partners and lacks clarity on distinguishing between harassment, sexual violence and marital rape (McKenzie, 2023).

DV is understood through various theoretical approaches, including social learning, feminist, evolutionary and exchange theories, and the social ecological model (Adames & Campbell, 2005; Rothman, 2018). Sociocultural factors strongly influence attitudes towards violence and gender norms (Song et al., 2021), emphasising the need to acknowledge cultural influences that impact gender norms embedded in societies. Culture significantly shapes individuals’ relationships with society (Yoshioka & Choi, 2005), influencing DV experiences. These norms intersect with other values, forming distinct perceptions of DV unique to each region. There is limited research of the cultural impact on DV in South American populations like Peru, where culture affects the frequency, characteristics, perception and societal response to abuse. Culture encompasses ideas shaping behaviour, categorising perceptions and labelling experiences through social transmission. Elements of culture, such as norms, ideologies, religion, laws and policies, vary greatly between regions (Mallory et al., 2016). Its importance is often overlooked in the literature.

Central aspects of culture include individualism versus collectivism. Every individual holds their own values, mindsets and perceptions. However, within a culture, certain values become more homogeneous and eventually predominant, shaping the societal norm (Fatehi et al., 2020). For instance, Western countries like the United Kingdom lean greatly towards individualism, while Peru exhibits collectivist values (Hofstede, 2011; Morales-Tristán & Rees, 2013).

In collectivist societies, cultural norms deeply influence individuals’ experiences, even if they personally reject the dominant values (Álvarez-Muelas et al., 2023; Peterson & Stewart, 2020). Conformity to group norms is rewarded, as those who adhere to these norms are viewed as more committed to collective ideals (Stamkou et al., 2019). Consequently, the needs of the group take precedence over individual desires, and the violations of norms elicit moral outrage, leading to feelings of anger and blame among both the group and external observers (Álvarez-Muelas et al., 2023; Stamkou et al., 2019). Decisions are typically made with the group in mind with strong emphasis on traditional family and gender roles, which is of utmost importance (Fatehi et al., 2020). Blame, shame and dishonour extend beyond the individual to all associated with them (Ashbourne & Baobaid, 2019). Non-adherence to family and gender roles is seen as a threat to the group’s honour (Ashbourne & Baobaid, 2019). The pressure to maintain social harmony and fulfil familial duties compels individuals to adopt various strategies, thereby creating an illusion of cultural uniformity (Álvarez-Muelas et al., 2023; Fatehi et al., 2020). Individuals may suppress and disregard non-dominant values to fulfil their social obligations, thus minimising conflict and their social standing (Fatehi et al., 2020).

In individualist cultures, people are perceived as unique and independent entities, distinct from others (Álvarez-Muelas et al., 2023; Fatehi et al., 2020; Peterson & Stewart, 2020). Individualist societies highly value independence, self-sufficiency, self-fulfilment, self-expression, privacy and freedom (Fatehi et al., 2020; Stamkou et al., 2019). As a result, self-interest and personal preference are prioritised to enable the achievement of these values (Fatehi et al., 2020). Research on Western cultures has shown the opposite effect on norm violators in terms of their social standing (Stamkou et al., 2019). Norm violators in individualist societies may increase their status when acting in terms of their own volition and needs, as this shows leadership, independence and power (Stamkou et al., 2019).

Independence, self-reliance and privacy are values that can inadvertently result in difficulties in seeking help and receiving help when facing DV (Pokharel et al., 2020). The expectation that individuals should be able to handle their own business, issues and circumstances places pressure on help seeking for victims out of fear of seeming weak and incapable of managing their circumstances independently (Örmon et al., 2016). Victim blaming ideologies towards coming forward or leaving an abusive relationship immediately (Örmon et al., 2016) can greatly discourage victims from seeking help if a narrative of responsibility for one’s own circumstances is reinforced due to a culture of personal strength, resilience and autonomy (Pokharel et al., 2020).

Gender power imbalances are entrenched in cultural and political spheres impacting DV (Shannon et al., 2017; Thompson & Figueroa, 2021; Tonsing & Tonsing, 2019). The UK has made considerable progress in domestic violence support services, public awareness, legislation and legal frameworks (Department for Levelling Up, Housing & Communities, 2024). Despite this, power imbalances and sexism remain ever present in the UK, affecting the gender disparities in DV (Sanders & Flavell, 2023; Women’s Aid, 2019). Revictimisation of women who have experienced DV continues to be of pressing concern despite efforts towards improved support services. Systemic gaps in legal protection measures and the poor conviction rates compared to the amount of reported DV cases exacerbate the issues, deterring those from seeking help (ONS, 2023). While gender imbalances in the UK persist, Peru has continuously held a culture of traditional gender norms rooted in distinct male and female roles (Bornstein et al., 2016; The World Bank, 2018).

The concept of ‘machismo’ is deeply embedded in Latin American culture, representing masculine pride, power, strength and aggression, contributing to power imbalances and high DV rates (Nuñez et al., 2016). Machismo encompasses a range of definitions and interpretations, with a common thread of virility, dominance and strength as a cultural expectation for manhood (Hernandez, 2003). It has both negative (aggression, sexual prowess, superiority) and positive (pride, honour, responsibility, courage) connotations (Hernandez, 2003). Peru’s specific definition of machismo implies that superiority and dominance over both men and women are seen as signs of a courageous man, while women are often viewed as coquettish, sexual and subservient (Justice Everywhere, 2020; Mamani-López et al., 2020; Scroope, 2018)

The impact of machismo has been clearly correlated to IPV regarding decision making and traditional roles outlined for the relationship. Machismo is a powerful predictor of violence considering it is rooted in aggression and asserting power over others (Canada: Immigration and Refugee Board of Canada, 2018; Mamani-López et al., 2020). This is because it refers to the cultural intrinsic belief on the dominance and strength of men, which inherently forms widespread gender violence as a result of distinct gender inequalities (Bozzi et al., 2022; Villa-Palomino, 2023). On the other hand, the definition most closely related to machismo in the UK is sexism. However, sexism is generally seen as discriminatory behaviour or attitudes based on gender, often manifesting in ways that perpetuate inequality and are condemned by society (Beetham et al., 2021; Treasury Committee, 2024).

Catholicism is a significant element of Peruvian collectivist culture. Those in religious communities often have larger social networks (Hawley et al., 2015). However, religion can also reinforce traditional gender and family roles, perpetuating gender inequalities (Green et al., 2024). ‘Marianismo’, a concept linked to the Virgin Mary, promotes submissiveness, chastity, traditional femininity, selflessness and specifically the acceptance of machismo (Green et al., 2024). Women adhering to Mariana traits may perceive silence and acceptance of male-inflicted suffering as positive, maintaining social harmony (Viana et al., 2022). In Latinx cultures, DV is often socially considered justified when behaviours conflict with traits of marianismo, such as infidelity in marriage (Bucheli & Rossi, 2019). The Catholic Church’s rejection of divorce can further endanger women, as religious leaders may encourage silence and maintaining their relationships with abusers, and advise continued unity of the family above the wellbeing needs of women (Castro et al., 2017; Green et al., 2024). Religion in the UK significantly impacts perceptions and responses to domestic violence, but its influence is less uniform than in Peru due to the UK’s religious diversity. In the UK, Christianity is the predominant religion, yet it accounts for less than half of the population (ONS, 2021). The country holds a variety of religious and non-religious communities, leading to a heterogeneous mix of beliefs, values and practices (ONS, 2021).

This religious diversity means that there is no single, widely accepted religious norm influencing attitudes towards domestic violence. Thus, religious teachings do not uniformly shape societal views on gender roles, forgiveness, reconciliation or family unity. The lack of a dominant religious framework results in a less cohesive impact on domestic violence, with various religious and secular groups providing different levels of support and intervention (Istratii & Ali, 2023).

Cross-national studies often focus on Western, educated, industrialised, rich and democratic (WEIRD) countries, adopting political or legislative perspectives (Knickrehm & Teske, 2000; Smartt & Kury, 2007). Cultural comparisons offer insights into public perceptions rooted in deeply ingrained societal ideologies.

## Method

### Design

This research seeks to compare conceptualisations and attitudes towards DV in the UK and Peru. The country selection arises from the intersection of the researchers’ cultural competence and the need to explore collectivistic versus individualistic cultures. While South American countries share many cultural commonalities, each country has its own distinct social and legal understandings and responses to domestic violence. The lead researcher has in-depth understanding of Peruvian culture, and both the lead and second researcher have a great understanding of the cultural experiences in the UK and the wider cultural experiences of South American countries. The understanding of complex cultural characteristics is essential in this research. The intersection of such knowledge establishes the cultural competence of the researchers in this area.

The research then aims to provide context for these differing attitudes through the qualitative conceptualisations of DV between Western and South American populations. The objectives of this study are to (a) recognise similarities and differences in the conceptualisation of DV, and (b) identify blame allocation differences using the Domestic Violence Blame Scale (DVBS) to inform cultural best practices within charity, government and medical sectors.

The analytic process involved adopting a cross-cultural mixed-methods exploratory design. Thematic analysis of qualitative data was performed to identify patterns, which were then coded into emerging themes (Braun & Clarke, 2006). Each question was analysed separately within each country (UK and Peru), and the results were then compared by question between the two countries. A similar approach was applied to the quantitative DVBS scale, where each question within the four DVBS sections was compared between countries. Blame attribution is often influenced by cultural and social norms; therefore this design reflects the aim to understand these factors with further qualitative context in efforts to inform culturally relevant frameworks that acknowledge and address the unique barriers faced by individuals from different backgrounds. This is especially important in multicultural societies where diverse beliefs and values can impact help-seeking behaviours.

### Sample

Participants were recruited using a convenience and snowballing sampling approach, facilitated by sharing digital links to a survey with a combination of open-ended and closed questions through online public social media platforms. To ensure participants’ complete anonymity, demographic factors were not collected, as the priory hypothesis was discerning cultural distinctions rather than socio-demographic attributes. While not a central aspect of the current study, future investigations may find value in exploring these factors. Eligibility criteria mandated participants to be over the age of consent in the UK and Peru (16 years of age), currently residing in the UK or Peru, and having lived in either respective country for a minimum of 16 years.

A priori power analysis was conducted using G*Power Version 3.1.9.7 (Mayr et al., 2007). The minimum sample size required for this study was determined to test the hypothesis of this study. The results of the G-power analysis indicated that a sample size of 40 was needed to achieve 80% power to detect a large effect size (*d* = 0.8; Cohen, 1988) at a significance level of .05 (Magnusson, n.d.). The obtained sample size of *n* = 41 was deemed adequate to test the hypothesis of the study.

This methodology is in accordance with cross-cultural and qualitative research, wherein studies characterised by smaller sample sizes have nonetheless yielded substantial and comprehensive insights into the subject area (Freed et al., 2017; Lysova et al., 2020; Zark & Satyen, 2022). The employment of a sample size of 41 participants in this study is sufficient for comparative analyses due to the exploratory nature of this cross-cultural research as well as its novelty. The design approach allows for in-depth exploration of cultural nuances and patterns to understand complex cultural differences within this sample size.

### Measures and materials

Participants completed a two-part online questionnaire, the first of which was a three-question open-ended section created for this study. These three questions were as follows: (a) ‘What is your definition of domestic violence?’; (b) ‘What behaviours do you associate with domestic abuse?’; and (c) ‘Who might be affected by this?’. To avoid potential bias, the qualitative section was placed before the DVBS in this study. This was done so that participants were not steered towards specific definitions of DV when answering the qualitative items considering the DVBS mainly outlines DV as male perpetration and physical violence.

After the completion of the qualitative items, the DVBS questionnaire developed by Petretic-Jackson et al. (1994) was presented. The DVBS is composed of four contexts of DV blame attribution (situational, perpetrator, societal, victim). The DVBS uses a 6-point Likert scale (1 = representing strong disagreement, 6 = representing strong agreement) for the assessment of participants’ level of agreement with each of these forms of blame. There are 5–7 multiple choice questions in each section and 23 questions total. The situational blame section assesses the degree to which respondents believe situational factors contribute to the occurrence of DV (e.g. financial issues, stress). The perpetrator blame section assesses the extent to which respondents believe the perpetrator holds blame for the occurrence of DV. The societal blame section assesses the degree to which respondents believe societal factors contribute to the occurrence of DV (e.g. social policies, cultural norms and values, legislation). The victim blame section assesses the extent to which respondents hold the victim responsible for the occurrence of DV. Individual items in each section are summed to create a total score for each of the four sections of the DVBS. A higher score indicates a higher level of blame attribution in the respective sections. Scores for each section are compared between groups to identify differences in the tendency to attribute blame to different contexts.

### Procedure

Ethical Approval was granted by the Health, Psychology, and Social Care College of the University of Derby (ETH2122-2075).

The invitation to participate was disseminated through public social media groups. All documents including participant invitations, information sheets, consent forms, the questionnaires and debrief forms were translated to Spanish for the Peruvian population. Participants were asked to answer three open-ended questions to the best of their ability. The second part of the questionnaire involved four sections made up of 23 questions total. Participants were given brief directions to mark their level of agreement (strongly disagree to strongly agree) for each question. Once they completed the questionnaire, they were provided with a debrief of the study, which involved greater detail of the study and links to related papers on perceptions of DV. Data collection did not consider rapport building between researcher and participant, as the data collection occurred online through questionnaires, and without interviews. The dataset was retrieved from Qualtrics. The quantitative data were analysed using SPSS IBM Statistics 27, and the qualitative data following the Thematic Analysis approach as described by Braun and Clarke (2006).

## Results

### Thematic analysis

A thematic analysis was conducted for each of the three questions in the qualitative portion of the questionnaire. The Peru and UK responses produced multiple themes, which were then compared.

#### Defining domestic violence

The first question, ‘What is your definition of domestic violence?’, revealed two main themes for the UK population and three for the Peruvian population.

In the UK population, domestic violence was defined in two parts. The first part is described as the type of violence that occurs and was mainly identified as physical and psychological violence (‘Physically violent behaviour and mental abuse’; ‘Emotional or physical abuse through deliberate manipulation or violence’). The first theme in the Peruvian respondents aligned with the UK respondents in terms of including the types of violence as a key component in defining domestic violence and also primarily described the type of violence as physical and psychological (‘Any act in which the physical or psychological peace of people is violated.’; ‘whether physical or psychological’).

The second theme in the UK’s definition of DV involved the relationship to the perpetrator, mainly viewed in the context of a romantic relationship between partners (‘Someone you are in a partnership with in some form’; ‘Being abusive to your partners’; ‘Violence committed by one person to another who are in a relationship’). Somewhat like the UK’s second theme, Peru also defined DV by who it involves. However, there is a key difference between the second themes as the interpersonal element included in both definitions was conceptualised by where it occurs and who it involves in Peru. Peru described DV as occurring ‘within the home’. The excerpts demonstrate that DV was defined by violence that takes place inside the home and involves anyone who resides in the residence (‘abuse within the family environment’; ‘It is violence within the home’; ‘abuse of a partner, towards a child, towards an older adult, within the home’). This can include romantic partners, children or grandparents/elderly if they reside in the home. Contrary to the UK, Peru produced a third theme when defining DV, which includes the impact of DV on the family (‘Affecting their integrity and dignity as a human being’; ‘It encompasses more than all that feeling of fear and feeling insecure in their own home, which should be the opposite of the concept of family, unfortunately, it happens and in Peru it is a country with high levels of family and machismo’). This part of the definition was described as a violation of peace, dignity, integrity and tranquillity of the home and family.

#### Behaviours associated with domestic violence

The second question, ‘What behaviours do you associate with domestic abuse?’, revealed psychological abuse as a main theme for both countries. However, psychological abuse is a broad description, and each country described psychological abuse in distinctly different manners. The UK mainly associated gaslighting as a form of psychological abuse (‘gaslighting, making them doubt their own convictions/believe they deserve this due to their own behaviour’; ‘emotional-gaslighting’; ‘mental-indoctrination’), while Peru mainly associated it with humiliation (‘putting someone down, ignoring someone’s voice’; ‘taking actions that humiliate another member’).

Controlling behaviour also emerged as a key theme in the UK, described as a restriction of freedom, including social and financial components (‘keeping them away from others like family’; ‘Controlling e.g. jealousy. Can’t go to X to see Y’; ‘Controlling, possessive, intimidating behaviour’). The UK population described physically violent behaviour as cuts, hitting, punching, kicking and pushing (‘physical violence from pushing, gripping, to outright smacking’; ‘hitting, punching’). Sexual violence was also included as a form of physical violence (‘physical violence like hitting and rape’; ‘physical beating, sexual assault, assault’).

Peru showed an emerging theme of verbal abuse, mainly insults and yelling or shouting (‘person who yells, insults’; ‘shouting, insults, bad comments if frequent’). Peru also associated physical abusive behaviour with DV, describing it as aggression with reference to superiority and ownership of women (‘The belief that your partner is an object that only belongs to you’; The person who believes he is superior for attacking another just for being stronger or manipulative in the home’). Peru’s final key theme was characterised as machismo rather than physical abuse, emerging from multiple references to machismo as an associated behaviour of DV and complexities in aggressive behaviour that hold elements of power imbalances and superiority between partners.

#### Those affected by domestic violence

The third question, ‘Who could be affected by domestic violence?’, received unanimous responses from both the UK and Peru, with both groups stating that ‘anyone’ could be affected. However, this response held distinctly different meanings. The UK responded to this stating any gender, age, race, level of education and partners at any stage of the relationship could be affected by DV (‘Anyone! Men, women, children’; ‘All genders, all ages, all stages of relationships’; ‘Anyone can be a victims of domestic violence disregarded of gender, race, sexuality’). On the other hand, the Peruvian population were concerned with ‘anyone’ affected within the domestic sphere (‘Any member of the family’; ‘In reality, all the members of the family are directly or indirectly affected’). While both the UK and Peru agree that ‘anyone’ can be affected by domestic violence, distinct differences in responses, where the UK emphasises a broader societal context while Peru focuses on familial dynamics, indicate distinct mindsets that can be culturally explained through societal contexts.

### Quantitative analysis of DVBS

The data were assessed for any violation of assumptions in normality, large sample size and the homogeneity of variance for the *t* tests. The normality assumption was met. There was homogeneity of variances between groups, as assessed by the Levene’s test for perpetrator responses (*p* = .132) but not situational (*p* = .035), societal (*p* = .001) and victim responses (*p* = .001).

An independent-measures *t* test was then conducted for the DVBS comparing the level of blame attributed to four contexts of DV blame between countries. The DVBS questionnaire contains four main topic sections, which can be analysed separately. For each of these sections, an independent-measures *t* test would allow a comparison in the mean level of agreement in blame attribution contexts between the two countries (Hole, 2009).

The DVBS had five to seven questions per section, therefore the data were analysed by averaging the scores of each of the four contexts for each country and comparing the scores of each context to the scores of the respective country as outlined in Bryant and Spencer’s (2003) DVBS study.

The 21 UK participants who responded to the DVBS (*M* = 3.61, *SD* = 1.2) were compared to the 20 Peruvian participants (*M* = 4.2, *SD* = 1.47) and demonstrated a significant difference in scores for the situational blame, *t*(203) = −3.11, *p* = .04. These results indicate there was a significantly high difference in situational blame scores between countries in which Peru demonstrated higher levels of agreement for situational blaming in DV than the UK (Table 1).

**Table 1.** Independent-samples *t* test equality of means.

	UK			Peru			*df*	*t*	*d*
	*N*	*M*	*SD*	*N*	*M*	*SD*			
Situational	21	3.61	1.2	20	4.2	1.47	203	−3.11*	1.34
Perpetrator		3.39	1.42		4.34	1.32	203	−4.95	1.37
Societal		3.14	1.42		4.1	1.17	203	−5.76**	1.3
Victim		1.45	0.82		1.84	1.28	285	−3.06**	1.07

*Note:* Independent-samples *t*-test was conducted measuring the difference in levels of agreement in four blame contexts for the UK and Peru.

*p < .01; **p < .001.

An independent-samples *t* test was conducted to compare level of blame agreement scores in perpetrator contexts for the UK and Peru population. The 21 UK participants who responded to the DVBS (*M* = 3.39, *SD* = 1.42) were compared to the 20 Peruvian participants (*M* = 4.34, *SD* = 1.32) and demonstrated a non-significant difference in scores for the situational blame, *t*(203) = −4.95, *p* = .13. These results indicate that the UK and Peru did not significantly demonstrate differing levels of agreement with perpetrator blame.

The comparison of the level of blame agreement scores in societal contexts for the UK and Peru population, between the 21 UK participants who responded to the DVBS (*M* = 3.14, *SD* = 1.42) and the 20 Peruvian participants (*M* = 4.1, *SD* = 1.17), demonstrated a significant difference in scores for the situational blame, *t*(244) = −5.76, *p* = .00. These results show that Peru demonstrated higher levels of agreement in blaming societal factors in DV than the UK.

The level of blame agreement scores in victim contexts for the UK and Peru population was also analysed. The 21 UK participants who responded to the DVBS (*M* = 1.45, *SD* = 0.82) were compared to the 20 Peruvian participants (*M* = 1.84, *SD* = 1.28), and it was evidenced that there was a significant difference in scores for situational blame, *t*(285) = −3.06, *p* = .00, thus indicating that Peru demonstrated higher levels of agreement for victim blaming.

## Discussion

This study aimed to investigate and compare the conceptualisations of domestic violence and attitudes towards DV in the UK and Peru. The DVBS questionnaire was utilised to identify the level of blame attributed to victim, perpetrator, societal and situational factors in the occurrence of DV. The thematic analysis focused on three aspects: the definition of domestic violence, the types of behaviours associated with it and the perception of who is affected by DV. In conjunction, the results reveal significant cultural differences in the conceptualisations, perceptions and attitudes towards DV between the two countries.

When defining DV, both countries identified psychological and physical abuse as primary types of violence. UK national statistics do typically report more incidents of psychological and physical violence, with lower reports of sexual violence aligning with the main perceived forms of violence described in the conceptualisation of DV in this study (Guedes et al., 2015; ONS, 2022; Women’s Rights Division of Human Rights Watch, 2000). IPV statistics for Peru show that psychological and physical abuse are the most prevalent forms of violence compared to sexual abuse rates (Barón-Lozada et al., 2022).

In Peru, the focus shifts to who within the family can be affected by DV rather than who in general society may be affected by DV. The Peruvian perspective considers anyone in the family as involved in DV including children, adults, grandparents and extended family members. This element of ‘where’ DV takes place indicates clear influences of a family-oriented mindset in Peru and aligns with their definition of DV, which characterises it as abuse within the family home, rather than solely between partners. Peru’s definition of DV also highlights its impact on family harmony, describing it as an infringement on family peace, dignity, integrity and tranquillity. This emphasis is particularly salient in Peru, where family harmony is highly valued due to large family sizes and collectivist values. Therefore, the legal definition and the perceived definition of DV in Peru, which includes the entire family, reflect the strong presence of collectivist mentality in the conceptualisation of DV.

Psychological abuse can take various forms, including intimidation, harassment, isolation and distortion of reality (Martinez-González et al., 2021). Notably, while both populations acknowledged psychological violence, UK respondents focused on psychological tactics like gaslighting. Gaslighting is a tactic employed to foster doubt and destroy one’s self-esteem and sanity to prevent the victim from recognising abuse and seeking help (Hightower, 2017; Sweet, 2019). While humiliation requires a public sphere to achieve an observed exertion of power, there is an inherent element of privacy in gaslighting, which aims to isolate and control. Perpetrators may gaslight their victim by insisting the victim is imagining or overreacting to the abuse while consistently trivialising their actions (Benjamin-Klein et al., 2023; Hailes & Goodman, 2023). This is particularly threatening in societies where individuals are expected to be self-reliant and independent. Members of individualist societies often have reduced community support and interconnectedness among family members. Despite the presence of barriers for DV victims in both the UK and Peru, the UK offers substantially more opportunities for formal support services through governmental and private organisations than does Peru. Consequently, the recognition of gaslighting as a fundamental psychological tactic in the UK can be explained through a cultural lens. Perpetrators may employ tactics of isolation and mental distortion to convince their victims that their experiences of abuse are acceptable and not deserving of the formal assistance available. These results are illustrative of the UK’s individualistic culture compared to the Peruvian, in which the public sphere assumes a wider role and relevance in Peru.

Peru’s emphasis on humiliation reflects the value of family dignity and social standing highlighting the impact of the public sphere in the conceptualisation of abusive behaviours. Humiliation is a manipulative strategy exerting power and control within the relationship (Leidner et al., 2012; Martinez-González et al., 2021). Ginges and Atran (2008) describe the outcome of humiliation as inertia, meaning victims feel they can neither reconcile with the perpetrator nor act out in aggression because of mixed feelings of outrage yet powerlessness. Humiliation in Peruvian culture is a powerful psychological tactic in domestic abuse, exploiting cultural values tied to family and gender roles. It targets the victim’s self-worth, leading to feelings of powerlessness, shame and a diminished sense of self. This impact is worsened by societal expectations that prioritise family unity over individual wellbeing, trapping victims in abusive situations to maintain the family unit. In a society where traditional gender roles are deeply ingrained, humiliation reinforces these power dynamics, making it even harder for victims to seek help. While the Peru Family Violence Law recognises humiliation as a form of psychological abuse (Agüero et al., 2018), it has been greatly normalised due to generations of familial socialisation under the guise of maintaining harmony (Martinez-González et al., 2021).

These cultural dynamics may be further illustrated by the societal blame scores, which emphasise the importance of maintaining family cohesion and adhering to traditional gender roles. The Peruvian population attributed more blame to the perception of women as property, the broader acceptance of male dominance and the societal acceptance of DV in marriage. The highly placed importance in maintaining the institution of marriage and the normalisation of traditional familial roles make it difficult to seek alternatives to enduring abuse. Women influenced by traditional Mariana values may endure abuse silently for the sake of family harmony (Fuchsel et al., 2016). Religious affiliation significantly affects the likelihood of DV victimisation (Seminario-Córdova & Paredes-Gutiérrez, 2021). Victims seeking help from the church may be steered towards conciliation, particularly if legal aid is unavailable. Legal perspectives in Peru also reveal an acceptance of violence in marriage, evident through the exclusion of marital rape and a preference for conciliation methods over legal action in abuse cases. This suggests that the role of societal structures in perpetuating DV, particularly through public reinforcement, has been widely normalised to prioritise gender and familial dynamics, even at the expense of individual wellbeing.

In the UK, physical violence was clearly identified as a key DV behaviour, while in Peru, it was described as aggression with elements of gender superiority, aligning with the concept of machismo. The responses indicated a strong consensus among participants that machismo, characterised by a sense of superiority and ownership, significantly manifests in domestic abuse in Peru. The higher blame attribution scores for victim and situational factors may reflect underlying gender superiority and contexts where gender imbalances contribute to increased blame attitudes. The high victim blame scores in Peru reflect the deeply ingrained belief that women should conform to certain behaviours and roles within the family, and failure to do so justifies violent repercussions. Respondents were more likely to believe that victims provoke or deserve DV, or that they exaggerate its effects. The main difference in victim-blaming scores may stem from the statement suggesting a ‘Rise in women’s movement caused domestic violence’. Literature suggests that non-Western countries tend to associate DV with lower socioeconomic backgrounds and lower education levels, in contrast to Western countries (Coll et al., 2020). Country-level increases in the proportion of highly educated women is typically a protective factor of IPV due to increased opportunity for economic independence and links to greater use of legal systems (Hardesty & Ogolsky, 2020; Klencakova et al., 2023). ; However, women with higher education levels than their partners in Peru are more likely to experience abuse, aligning with exchange theory, which posits that abuse is used to assert power and control when male dominance is threatened by traditional gender norms (De Waardt & Ypeij, 2017; Garay et al., 2022). Additionally, feminist movements in Peru have shown that women involved in grassroots organisations experience higher levels of abuse than those who are not (De Waardt & Ypeij, 2017).

The differences in the understanding of physical violence may also be framed through an understanding of unique situational differences impacting blame attitudes. The higher scores in situational blame among the Peruvian population suggest a greater tendency to attribute DV to external and contextual factors. In the situational blame section, participants were asked about blame attributed to poor and unstable homes, the use of drugs and alcohol, and social isolation. Decreased levels of agreement on domestic tasks such as cooking, cleaning, sex and affection can increase the possibility of IPV in such relationships where these are expected (Franklin et al., 2012). Women living in cohabitation with their partners are 1.14 times more likely to experience domestic violence than married women (Castro et al., 2017). This emphasis on cohabitation may be due to lower levels of commitment, uncertain relationship expectations or an increased likelihood of infidelity (Castro et al., 2017). These uncertainties in cohabitation without marriage infringe on the ideologies of machismo and traditional family or partnership roles in Peru. Partner alcohol consumption is a key risk marker in Peru, as women with alcohol-consuming partners are more than twice as likely to experience abuse (Castro et al., 2017; Seminario-Córdova & Paredes-Gutiérrez, 2021). Social isolation is another critical concern due to limited accessibility to government support agencies like Centres for Emergency Assistance for Women (CEMs), mainly situated in urban areas. This scarcity of support services, particularly in rural areas, forces victims to develop coping mechanisms or rely on informal networks or religious institutions that may advocate for reconciliation. The emphasis on situational blame may reflect the belief that DV is a product of broader social and economic conditions, rather than solely the actions of the individuals involved. In contrast, UK respondents placed less emphasis on situational blame, which could be indicative of the more individualistic cultural values prevalent in the UK, where personal responsibility and agency are prioritised over contextual influences. Participant perceptions confirm the unique situational barriers present between countries.

The final qualitative question demonstrated clear differences in the context of who can be affected by DV. Both countries agreed that ‘anyone’ can be affected by DV; however, the UK emphasised broader societal contexts while Peru focused on familial dynamics. The UK conceptualises those involved in domestic violence through a demographic lens, recognising that anyone can be susceptible to it, irrespective of gender, age, ethnicity, education or relationship stage. The UK defined DV based on the relationship between the victim and the perpetrator, aligning with its legal definition, which excludes children under the age of 16 and classifies such cases as child abuse. Consequently, it is fitting to describe the perception of DV in the UK as primarily associated to romantic partners. Inherently, family law is juxtaposed to this concept and legally signifies the involvement of all family members regardless of whether romantic partners are involved. This legal framework is supported by responses that highlight the need to address domestic violence as a collective familial issue. The UK reflects a more individual-focused mind frame within a broader societal context. This is not to say that the UK is not concerned with family violence, but instead that other relationships and their dynamic might be perceived as more central to domestic violence. Even though all disseminated materials used the wording domestic violence, in both English and Spanish, only Peru addressed strong family components when conceptualising DV. This is largely due to the legal definitions of domestic violence and the culture values, which go hand in hand to account for the difference in responses.

Both Peru and the UK agree that perpetrators are to blame for DV. Regarding perpetrator blame, both countries show similar levels of agreement that perpetrators should be held accountable, are often mentally ill and may have experienced violence themselves. Participants in both countries attribute DV to psychological issues that hinder behavioural control. In lower income countries like Peru, DV is often linked to mental illness in both perpetrators and victims, reflecting beliefs about specific personality traits and mental health issues (Coll et al., 2020). Conversely, higher income countries such as the UK focus more on learned behaviour and psychological problems, with a belief that violent perpetrators often had violent fathers (Coll et al., 2020). The social learning theory supports this view, where witnessing parental violence often leads to perpetuating or accepting violence in future relationships (Castro et al., 2017; Chernyak et al., 2020; Coll et al., 2020).

### Limitations

The DVBS focuses on physical forms of violence and male perpetration in DV contexts. To provide further considerations in DV conceptualisations, the qualitative portion firstly allows participants to provide a more holistic view of their perception of DV that may not necessarily align with the physical violence described in the DVBS. The underpinnings of this research are centred on the impact of culturally differing attitudes on DV. As a result, broader scale investigations encompassing several socio-demographic variables such as age, gender, education and location would be of value in understanding the multifaceted attitudes and conceptualisations of DV. Furthermore, the sample size should be widened in a follow-up study, allowing richer data and a more generalisable dataset.

### Implications for future frameworks

Formal services play a crucial role in providing risk management, safety planning and pathways to recovery (Green et al., 2024). These services can enhance outcomes by mitigating post-trauma effects and improve overall wellbeing for the entirety of the family (Green et al., 2024). Thus, addressing the cultural aspects of domestic violence requires tailored multifaceted frameworks that resonate with diverse populations (Fuchsel et al., 2016; Tsapalas et al., 2021; Tudela-Vázquez, 2015). This is because the sociocultural background may shape victims’ and survivors’ understanding of domestic violence, as results suggested, and likely their engagement with the support available. The articulation of their experiences within a meaningful cultural framework is then paramount (Ashbourne & Baobaid, 2019). For example, in the British context, there might be a higher focus on offender punishment and separation, whilst Peruvian victims and survivors might wish for a solution nearer conciliation or restoration. In fact, migrant women in the Western countries frequently report the need to conform to mainstream approaches such as reporting violence, or there could be a risk of losing service support (Green et al., 2024). It could also be that women from collectivist cultures are less likely to seek formal assistance, influenced by their community’s attitudes towards domestic violence (Ashbourne & Baobaid, 2019). On the other hand, the lack of understanding about formal support services due to recent migration may lead to increased reliance on informal support. This reliance is often based on the expectation that formal services will reflect patriarchal structures (Green et al., 2024), which might not be necessarily true. Furthermore, the length of stay in a new country can greatly influence help-seeking behaviour, where shorter stays predict higher likelihood to access informal help rather than formal help (Gonçalves & Matos, 2020). Understanding the intricate relationships between family dynamics, marriage, religion, policy and legislation is crucial for effectively addressing domestic violence among Latinx victims in the UK, as well as in South America.

According to Pan et al (2006) culturally relevant interventions could use native languages and emphasise restoration of the family harmony over combating domestic violence. For collectivistic populations, where family is paramount (Scroope, 2018), interventions could encompass entire families rather than focusing solely on individual partners, prioritising practical solutions that ensure family welfare. An example would be offender interventions paired with victim and family interventions. Such programmes should target cultural gender norms, models and inequalities, such as the ones promoted by machismo. It is also fundamental to raise awareness to the general population on DV, as well as to train police forces, support service workers and health professionals to be sensible and aware of such cultural intricacies. Such considerations are fundamental to increase service access, to improve the provision design and to empower victims into survivors.

In the UK, DV support services for Latinx women remain limited, despite recognition of the distinct social and cultural barriers they face. The UK government website provides key information in Spanish when users search for domestic violence or family support. The primary resources available are the national DV hotline and the Women’s Aid directory, which lists DV services by location (Home Office, 2018). While this tool offers a wide range of DV support services across the UK, specialised services that cater to the multifaceted needs of the Latinx community are extremely limited and often not accessible in Spanish. Language barriers are particularly problematic for migrant women who may not have participated in English for Speakers of Other Languages (ESOL) courses upon immigrating to the UK. Latinx women seeking therapeutic interventions, legal guidance and family services that align with their social, family or religious values may encounter specific challenges in accessing appropriate support as a result.

Latin American Women’s Aid (LAWA) provide culturally informed, multilingual services, including emotional support, legal advice and safety planning, tailored to the needs of Latin American women. A large proportion of LAWA’s clients are migrants who primarily require Spanish-language services to access counselling and legal guidance effectively (LAWA, 2023). Migrants who are on spousal visas or do not have an immigration status may find it difficult to seek help, especially if there is not support that is easily accessible in Spanish. Latinx migrants who live with the perpetrator in asylum supported accommodation are entitled to safe alternative accommodation options; however, the location of alternative accommodation options is dependent on availability and therefore may impact the accessibility to culturally relevant DV support should an individual need to relocate (Home Office, 2022). Accessibility of Latinx services like LAWA is limited in locations around the UK, and while some offer reach-in support (LAWA, 2023), this does not always constitute safe outcomes for migrants who have limited accommodation options outside of asylum supported accommodation, without recourse to public funds (NRPF), or the right to work. While asylum seekers have some options for safe accommodation if they are victims of DV, these options are contingent on their understanding of UK laws and entitlements, which may be challenging for those unfamiliar with the language and legal system. Therefore, accessible DV support services tailored to Latinx women are critical, particularly for migrant women facing compounded challenges in seeking help.

A search for family-oriented DV support programmes in both English and Spanish reveals a limited number of initiatives that adopt a ‘whole family’ approach to addressing domestic violence. Services such as Independent Domestic Abuse Services (IDAS) and NSPCC Learning have implemented family-based programmes, including ‘Whole Family’ and ‘Domestic Abuse, Recovering Together’ (DART). These programmes engage both parents and children through workshops and individual sessions (IDAS, 2024; NSPCC, 2021). They aim to mitigate the impact of abuse on the family by rebuilding mother–child relationships, improving parental self-esteem and reducing behavioural and emotional difficulties in children. Reported barriers to the effectiveness of the DART programme is the continued contact between the mother or child and the perpetrator (NSPCC, 2021). This need for separation may present challenges when working with Latinx communities where strong family ties are integral to the cultural fabric. For Latinx families, frameworks that necessitate clear separation from the perpetrator may be difficult to adopt, as this can lead to the separation of the entire family or even the individual’s broader community.

Additionally, the findings of this research indicate that Peruvian populations are more likely than the UK population to attribute domestic violence to situational, victim and social factors. Therefore, a more comprehensive approach should be considered when addressing DV within Latinx communities in the UK. Few programmes consider these external factors beyond the perpetrator that could help prevent reoccurrence or future abuse. Safe Lives, for example, employs a ‘Whole Picture’ approach to DV, which follows a four-tiered model aimed at ending abuse by addressing the whole individual, the whole family, the whole community and the whole society (SafeLives, 2021). This approach not only provides practical support, such as refuge and outreach services, but also engages in broader community and research strategies. These include addressing masculinity among men and boys, promoting awareness of healthy relationships within communities, and raising awareness of DV in schools and workplaces (SafeLives, 2021). This model may more closely align with the broader conceptualisation of DV within Latinx populations, as reflected in the study’s DVBS results.

While there are services that include family approaches and address external factors for DV outside of the perpetrator, these programmes remain limited in several ways when addressing the needs of Latinx populations in the UK. Some services that adopt a family approach are limited in location (LAWA, 2023; NSPCC, 2021), while other DV services have partnerships with several local professionals, agencies and organisations such as the NHS to implement their family programmes across the UK (IDAS, 2024). It should be noted, however, that the DART and Whole Family programmes do not mention the inclusion of family beyond a mother–child relationship. Western frameworks on family typically focus on the nuclear family, consisting of parents and children, whereas Latinx families are often larger and include extended family members, such as grandparents, siblings and cousins. Thus, while the ‘whole family’ approach may be appropriate within UK definitions of family, it may not fully address the needs of Latinx populations.

The ‘Whole Picture’ model may be able to offer a more holistic approach to meeting the needs of Latinx women by addressing DV beyond the immediate family. However, the model is not explicitly designed for non-English speakers and may not fully consider the cultural values and norms of Latinx women. While the model does mention comparing demographic information to ensure that services are accessible to all groups within the ‘whole community’ aspect, there is limited information on whether Latinx communities are effectively reached through this model.

## Conclusion

Culture is the system of beliefs, values, customs and knowledge transmitted through a range of practices and traditions across generations, demonstrated in daily behaviours and practices (American Psychological Association, APA, 2020). This study contributes to Western frameworks by offering culturally relevant insights into DV perceptions in the UK and Peru. The lack of cross-cultural research has left a knowledge gap for effective UK frameworks that consider cultural distinctions. This study explored DV conceptualisations, highlighting cultural influences related to family and machismo, along with legal influences from family laws and history policies on conciliation. It also assessed differences in blame attribution for situational, societal, perpetrator and victim contexts using the DVBS, aligning with the thematic analysis. In summary, the findings suggest that cultural factors such as individualism versus collectivism, machismo and societal norms play a critical role in shaping how DV is understood, who is blamed and how victims respond to abuse. Understanding these cultural differences is crucial for developing effective interventions and support services that are culturally relevant to the needs of Latinx populations in the UK.
